# Changes in self-rated health and quality of life among Syrian refugees migrating to Norway: a prospective longitudinal study

**DOI:** 10.1186/s12939-020-01300-6

**Published:** 2020-10-27

**Authors:** Jasmin Haj-Younes, Elisabeth Marie Strømme, Jannicke Igland, Bernadette Kumar, Eirik Abildsnes, Wegdan Hasha, Esperanza Diaz

**Affiliations:** 1 Department of Global Public Health and Primary Care, University of Bergen, PO Box 7800, 5020 Bergen, Norway; 2 Unit for Migration and health. Norwegian Institute of Public Health, PO Box 222 Skøyen, 0213 Oslo, Norway; 3 Department of Psychosocial Health, University of Agder, PO Box 422, 4604 Kristiansand, Norway

**Keywords:** Self-rated health, Quality of life, Refugees, Transients and migrants

## Abstract

**Background:**

Forced migrants can be exposed to various stressors that can impact their health and wellbeing. How the different stages in the migration process impacts health is however poorly explored. The aim of this study was to examine changes in self-rated health (SRH) and quality of life (QoL) among a cohort of adult Syrian refugees before and after resettlement in Norway.

**Method:**

We used a prospective longitudinal study design with two assessment points to examine changes in health among adult Syrian resettlement refugees in Lebanon accepted for resettlement in Norway. We gathered baseline data in 2017/2018 in Lebanon and subsequently at follow-up one year after arrival. The main outcomes were good SRH measured by a single validated item and QoL measured by WHOQOL-BREF. We used generalized estimating equations to investigate changes in outcomes over time and incorporated interaction terms in the models to evaluate effect modifications.

**Results:**

In total, 353 subjects participated in the study. The percentage of participants reporting good SRH showed a non-significant increase from 58 to 63% RR, 95%CI: 1.1 (1.0, 1.2) from baseline to follow-up while mean values of all four QoL domains increased significantly from baseline to follow-up; the physical domain from 13.7 to 15.7 B, 95%CI: 1.9 (1.6, 2.3), the psychological domain from 12.8 to 14.5 B, 95%CI: 1.7 (1.3, 2.0), social relationships from 13.7 to 15.3 B, 95%CI: 1.6 (1.2, 2.0) and the environmental domain from 9.0 to 14.0 5.1 B, 95%CI: (4.7, 5.4). Positive effect modifiers for improvement in SRH and QoL over time include male gender, younger age, low level of social support and illegal status in transit country.

**Conclusion:**

Our results show that good SRH remain stable while all four QoL domains improve, most pronounced in the environment domain. Understanding the dynamics of migration and health is a fundamental step in reaching health equity.

**Supplementary information:**

**Supplementary information** accompanies this paper at 10.1186/s12939-020-01300-6.

## Introduction

We are witnessing a time in which forced migration is surging and the need to ensure protection, health, and wellbeing of people on the move is ever so vital. This sentiment is echoed in the Sustainable Development Goals of *leaving no one behind* [1]. In every stage of the migration process (pre-migration, during migration and after resettlement) impact on health and wellbeing is inevitable [2, 3]. Possible health risks and potential protective factors influence the health outcomes of the migrant, and there is an ongoing attempt to identify the relevance of each of these factors [4].

Populations at risk of poor health and health care disparities are generally considered as being vulnerable [5]. Migrants may encounter several barriers to health care because of their legal status and due to economic and social marginalization. Forced migrants differ from other types of migrants in that they are survivors of persecution, violence, and war - factors that might add to their health vulnerability. Hence, it remains unclear if the selection described in the ‘healthy migrant effect’ that postulates migrants’ health advantage compared to both citizens in the home country and in the host country holds true for refugees and other forced migrants [4, 6]. The accumulation of stressors leading to deterioration in migrants’ health over time have been explained by the ‘exhausted migrant theory’ [7]. Others have suggested that the migration experience in itself could be the cause of this deterioration [8] addressing the very act of migration as a social determinant for migrants’ health [9].

Despite forced migrants’ exposures to stressful events, there is also increasing evidence of positive mechanisms like post-stress growth, described as positive changes following adversity [10], and resilience, which is characterized by the ability to exhibit a stable health trajectory in difficult times [11]. Consequently, both adverse conditions rendering forced migrants susceptible for health disparities and the sources of resilience and growth must be considered in attempting to understand migrant health [12]. Furthermore, these factors need to be understood in synergy with contextual factors as well as embedded in a life trajectory, highlighting the different migration stages [13].

Although the body of evidence in terms of morbidity and mortality of migrants in host countries is growing, research on forced migrants throughout their often long journeys continues to be scarce [4], and has largely been limited to cross-sectional designs [14]. Also, previous research on forced migration has focused mainly on mental health [2, 15], often centered on negative health outcomes, predominantly in torture and trauma victims. Knowledge of overall and general health in non-clinical refugee populations remains insufficient.

Self-rated health (SRH) has proven to be a valuable predictor of all-cause mortality and morbidity [16, 17], including in minority populations [2], and is widely used in health monitoring and to research health inequalities. Quality of life (QoL) is considered a fundamental construct in public health that reflects complete wellbeing, going beyond old paradigms viewing health as merely the absence of disease [18].

Migration is a global, multifaceted, and dynamic phenomenon in which the migration experience in itself constitutes an important segment of the health trajectory [8, 9, 13]. In line with recommendations to address multiple phases of the migratory process [3] we aimed to assess general health among Syrian refugees following their health trajectory from a transit setting to after resettlement using a salutogenic approach. Specifically, our research questions are: 1) how does SRH and QoL of forced migrants change from the transit phase to the early resettlement phase? 2) Which factors (sociodemographic, social support, and migration related) can be identified as modifiers of change? As a second aim, we sought to compare our participants QoL-scores with international samples of QoL used as reference points against which we can interpret our findings. We hypothesized that our cohort of forced migrants would have a stable or decreasing health status after resettlement, as a consequence of post-migration stressors such as acculturation stress, poor access to healthcare, cultural discontinuity, loss of social support and perceived stigma and discrimination [2, 3, 19].

## Methods

Data for this study were from the CHART project (*Changing health and healthcare needs among the Syrian refugee trajectory to Norway* [20]), designed with a trajectory perspective to investigate refugee health over time. The reporting follows the STROBE statements for cohort studies.

### Study design and participants

This is a prospective longitudinal study assessing adult Syrian refugees under the UNHCRs international protection mandate admitted for resettlement to Norway at two time points. Baseline measures were gathered through a self-administered survey in Arabic in Lebanon between August 2017 and April 2018 in collaboration with the International Organization for Migration (IOM). Inclusion criteria were Syrian nationals from 16 and above attending mandatory pre-departure educational activities in the given time period, a total of 514 persons. Exclusion criteria were unaccompanied refugee minors between 16 to 18 years and severe mental disorder. However, no one was excluded based on mental health. The questionnaire was distributed during class time under the supervision of trained bilingual staff with cultural competence, available to assist persons with low health literacy, illiteracy or low Arabic language proficiency, and to pick up signs of mental distress in case of re-traumatization. Participants were compensated with the approximate equivalent of $10 USD after completing the baseline questionnaire. After arrival in Norway, the study participants were settled in 134 different municipalities throughout the country. Hence, follow-up measures were gathered through telephone interviews by Arabic-speaking study personnel. The Norwegian Directorate of Integration and Diversity and the municipalities’ immigration units provided contact information for the participants after resettlement. A total of 506 eligible subjects were accepted to participate (98%) in the study at baseline, out of whom 464 (92%) were confirmed resettled in Norway and 353 of 464 (76%) followed-up (Additional file 1).

### Dependent variables

In this study, we use two indicators for health as main outcomes: SRH and QoL. We have applied a salutogenic approach that is reflected in the selection and categorization of variables.

### Self-rated health

As a proxy for general health, SRH was assessed using the single-item question: How do you consider your health at the moment? This question is answered using a five-point response scale from very poor to very good. The item was dichotomized into a binary measure distinguishing between Good and Very Good compared with Very poor, Poor and Neither. The SRH measure has shown reliability and validity among Arabic speakers and within refugee populations [14, 21].

### Quality of life

QoL was measured using the WHO Quality of Life Scale (WHOQOL-BREF). The WHOQOL-BREF was selected because it was developed as a transcultural instrument and has demonstrated good psychometric properties, reliability, and validity among Arabic speakers [22]. The instrument comprises 24 items measuring four domains; physical health (seven items), psychological health (six items), social relationships (three items) and environment (eight items). The physical health domain entails questions on pain, medical treatment, energy, sleep, mobility and capacity. The psychological domain includes questions on concentration, self-esteem, meaningfulness and positive and negative feelings and thoughts. The social domain focuses on satisfaction with relationships, practical social support and sex-life. The environmental domain pertains to questions on safety and security, access to healthcare, financial recourses and physical environment. Each item is rated on a 5-point Likert scale with a higher score denoting a better QoL on the corresponding domain. Raw scores were transformed creating domain scores within the range of 4–20 by multiplying the average of the items in each domain by four, in accordance with instructions from the manual. Cronbach’s alpha for the total scale for the present sample is 0.8.

### Independent variables

#### Sociodemographic variables

The questionnaire included sociodemographic variables such as age, gender, mother tongue, marital status, number of children and years of schooling. We also inquired on migration related factors such as time since flight from Syria, time since arrival in Lebanon, number of transit countries before arriving in Lebanon, migrating alone or with family, and residence permit in Lebanon. In addition, we assessed Health Literacy through the single-item literacy screener (SILS): “How often do you need help reading written material from your doctor or pharmacy?” Possible responses are: Never (1), Rarely (2), Sometimes (3), Often (4), and Always (5). Scores higher than 2 point to difficulties with reading health-related material. We created a binary measure and used the variable *high health literacy* defined as responses ≤2.

### Social support

Perceived social support was measured with The ENRICHD Social Support instrument (ESSI), a short validated self-report measure that assesses the four defining elements of social support: emotional, instrumental, informational, and appraisal with 7 items [23]. A total score is the sum of all items with higher scores indicating better social support. We created a binary measure for high social support defined as having answered > 2 on at least two of the seven items and a total score of > 18 based on the definition of low-social support by the ENRICHD investigators [23]. ESSI has previously been validated among Syrian refugees [24]. Cronbach’s alpha for the present sample is 0.85.

Questions not already validated, such as demographic questions and migration related questions went through a translation process based on the ISPOR principles of good practice guidelines [25]. We included the following steps; two independent forward translations, reconciliation of the forward translation into one translation, back translation, harmonization, cognitive debriefing among a group of 6 respondents and proof reading.

### Statistical analysis

Descriptive data were presented as frequencies and percentages for categorical variables and as median with inter-quartile range (IQR) for continuous variables. Sensitivity analyses between the participants and the loss to follow up group were conducted using *χ*^2^-statistics and independent group’s *t*-tests. We analyzed the longitudinal data using generalized estimating equations (GEE) in long format with “wave” as a binary covariate to evaluate change in outcome from baseline to follow-up. The GEE method accounts for the non-independence of repeated data from the same subject. For binary outcomes we applied a log-link and binomial distribution and reported exponentiated regression coefficients as risk ratios (RR) with 95% CI. For continuous outcomes we applied an identity link and Gaussian distribution and reported regression coefficients (B) with 95% CI. To view our results in relation to other populations, we presented mean values of the WHOQOL-BREF domains together with mean values from Skevington et al. [26]. Their research is based on a sample of 11,830 adults from 23 countries across the globe, including Norway. We compare our sample with both the total sample of 11,830 subjects as well as with only the Norwegian sample of 1047 subjects, separately. To evaluate potential effect modifiers for change in outcomes over time we stratified by various characteristics measured at baseline in Lebanon (gender, age, ethnicity, marital status, education, level of health literacy, level of social support (ESSI), time in transit, multiple transit countries, residence permit in Lebanon, migrating alone) and incorporated interaction terms between the covariates and wave in the GEE models to test for significant differences in change over time for different subgroups. Missing values were handled through list wise deletions. An alpha value of 0.05 was considered statistically significant. We analyzed the data using STATA/IC software, version 15.1, (StataCorp LLC, Texas, USA).

## Results

A total of 353 subjects completed both assessments (baseline and follow-up) resulting in an attrition rate of 24% (Additional file 1). The most common reasons for loss-to-follow-up from Lebanon to Norway was not answering the phone/unreachable after a minimum of three attempts and declining participation. Apart from higher health literacy among respondents (56% versus 45%), no statistically significant differences in characteristics were seen between responders and non-responders (Additional file 2).

### Demographics at baseline

The overall median age of the cohort was 34 years (IQR 27–41), and 49% were males (Table 1). Participants had an average of 8 years of schooling and three out of four respondents were married (75%). Most of the participants had been migrants for approximately five years at baseline. A majority had high health literacy (56%) and approximately one third (35%) had high social support.

**Table 1:** Sociodemographic and migration related factors at baseline, *N* = 353

SOCIODEMOGRAPHIC FACTORS		
Gender (n, %)		
Women	181	51
Men	171	49
Age in years (median, IQR)	34	27–41
Mother tongue (n, %)		
Arabic	335	95
Kurmanji	15	4
Marital status (n, %)		
Married	265	75
Living with partner among married	260	98
Number of children (median, IQR)	4	3–5
Education in years (median, IQR)	8	6–10
High health literacy^a^ (n, %)	195	56
High social support^b^ (n, %)	123	35
**MIGRATION RELATED FACTORS**		
Time since flight from Syria at baseline in years (median, IQR)	5	4–6
Time since arrival in Lebanon at baseline in years (median, IQR)	5	4–5
Been in other transit country before Lebanon (n, %)	20	6
No residence permit in Lebanon at baseline (n, %)	242	69
Migrating alone to Lebanon (n, %)	55	16

^a^High health literacy defined as scores ≤ 2. ^b^High social support defined as > 2 on at least two of the seven items and a total score of > 18

### Changes in health from baseline to follow-up and comparison to other populations

Table 2 presents the main outcomes at baseline and follow-up. More than half of the respondents rated their health as good at baseline with a non-significant increase at follow-up RR, 95%CI: 1.1 (1.0, 1.2), *P* = 0.072. In the QoL domains, the highest domain scores were observed in physical health and in social relationships. Both domains showed a statistically significant increase at follow-up from 13.7 to 15.7 B, 95%CI: 1.9 (1.6, 2.3) and from 13.7 to 15.3 B, 95%CI: 1.6 (1.2, 2.0), respectively. The lowest scores at baseline were observed in questions relating to the environment followed by the psychological domain but these also increased at follow-up, from 9.0 to 14.0 B, 95%CI: 5.1 (4.7, 5.4) and from 12.8 to 14.5 B, 95%CI: 1.7 (1.3, 2.0), respectively. Overall, all the QoL scores were significantly higher in the follow-up assessment.

**Table 2:** Changes in prevalence (%) in dichotomous outcome (SRH) and mean (SD) score for continuous outcome (WHOQOL-BREF four domain scores, range 4–20) from baseline to follow-up, *N* = 353

	**Baseline**		**Follow-up**		**Change**	***P*-value**
**Self-rated health**	**N**	**n (%)**	**N**	**n (%)**	**RR (95% CI)**	
Good SRH	349	203 (58)	351	222 (63)	1.1 (1.0, 1.2)	0.072
	**Baseline**		**Follow-up**		**Change**	
**Quality of life (WHOQOL-BREF)**	**N**	**Score (SD)**	**N**	**Score (SD)**	**B (95% CI)**	
Physical health (Domain 1)	353	13.7 (2.7)	353	15.7 (2.8)	1.9 (1.6, 2.3)	< 0.001
Psychological health (Domain 2)	353	12.8 (2.7)	353	14.5 (2.3)	1.7 (1.3, 2.0)	< 0.001
Social relationships (Domain 3)	353	13.7 (3.0)	352	15.3 (2.8)	1.6 (1.2, 2.0)	< 0.001
Environment (Domain 4)	353	9.0 (2.4)	353	14.0 (2.2)	5.1 (4.7, 5.4)	< 0.001

Abbreviations: *RR* Relative risk. *CI* Confidence interval. *SD* Standard deviation

**Fig. 1:**
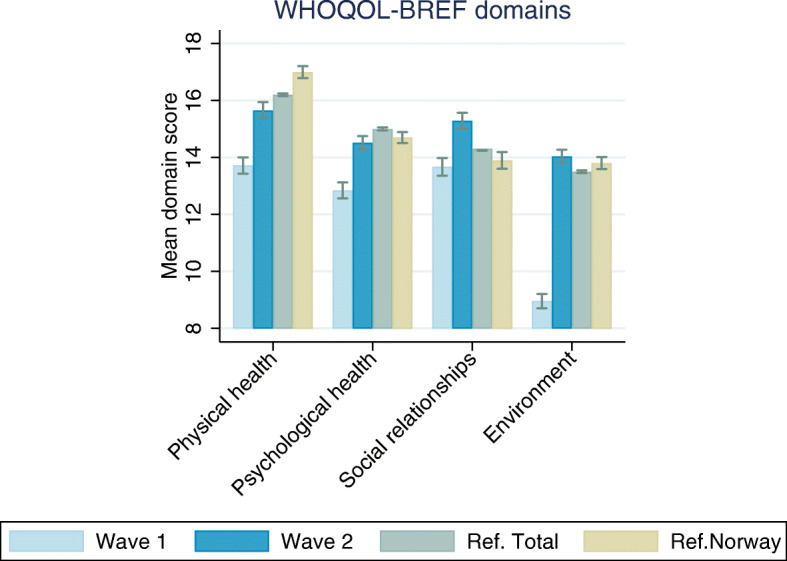
Mean (SD) score for continuous outcome (WHOQOL-BREF four domain scores, range 4–20) from baseline to follow-up compared with data from the WHOQOL-BREF field trials from Skevington, Lofty & O’Connell (2004). Error bars represent 95% CI

In Fig. 1, we compare changes in mean values with data from the international field trials of the WHOQOL-group, using both the sum of all field countries’ mean QoL-scores as well as Norwegian QoL-scores as reference points, separately [26]. At baseline, mean values for the physical, psychological and environmental domains were significantly lower than both international and Norwegian reference scores but improved to nearly the same levels at follow-up. The social relationship domain matched the international and Norwegian reference scores at baseline and surpassed these levels at follow-up.

### Effect modifications

Risk ratios and regression coefficients from stratified models with test for effect modification are reported in Table 3. We found a statistically significant improvement in the proportion of good SRH among men, but not among women, with a statistically significant interaction effect by gender. The improvement in social relationships (domain 3) and environment (domain 4) was also significantly larger in men. We also observed an interaction by age in the psychological domain (domain 2) with statistically significant improvement only among participants < 40 years of age. For marital status, the only statistically significant interaction was observed in the environmental domain (domain 4), with larger improvement among those who were married. When stratifying on level of social support (ESSI), there was a significantly stronger improvement among those with low social support at baseline in SRH and in the psychological and environment domain (domain 2 and 4). In terms of having a residence permit in Lebanon or not, statistically significant improvement in good SRH and social relationships (domain 3) was seen only among participants with no residence permit at baseline.

**Table 3:** Effect modification of change in dichotomous outcome (SRH) and continuous outcomes (four domains of WHOQOL-BREF) by selected sociodemographic and migration-related variables using interaction terms in generalized estimating equations, *N* = 353

	Good SRH		Physical health		Psychological health		Social relationships		Environment	
	RR (95% CI)	P	B (95% CI)	P	B (95% CI)	P	B (95% CI)	P	B (95% CI)	P
**Gender**										
Male	1.2 (1.1, 1.4)		2.2 (1.7, 2.7)		1.9 (1.3, 2.4)		2.1 (1.5, 2.6)		5.5 (5.0–5.9)	
Female	1.0 (0.6, 1.1)		1.7 (1.2, 2.1)		1.5 (1.1, 1.9)		1.2 (0.6, 1.7)		4.7 (4.2, 5.1)	
Interaction test		0.04*		0.157		0.293		0.027*		0.023*
**Age**										
< 40 years	1.1 (1.0, 1.2)		2.1 (1.8, 2.5)		1.9 (1.5, 2.3)		1.6 (1.2, 2.1)		5.1 (4.7, 5.5)	
≥ 40 years	1.1 (0.9, 1.4)		1.3 (0.5, 2.1)		0.9 (0.1, 1.6)		1.4 (0.7, 2.1)		5.0 (4.3, 5.7)	
Interaction test		0.793		0.056		0.016*		0.533		0.677
**Marital status**										
Married	1.1 (1.0, 1.3)		2.0 (1.6, 2.5)		1.8 (1.4, 2.2)		1.5 (1.0–1.9)		5.2 (4.9, 5.7)	
Other	1.0 (0.9, 1.1)		1.6 (1.0, 2.2)		1.2 (0.6, 1.9)		2.1 (1.3, 2.8)		4.5 (3.8, 5.2)	
Interaction test		0.478		0.243		0.121		0.202		0.048*
**High social support (ESSI)**										
Yes	0.9 (0.8, 1.1)		1.5 (0.8, 2.1)		0.8 (0.2, 1.4)		1.4 (0.8, 1.9)		4.4 (3.9, 5.0)	
No	1.2 (1.1,1.3)		2.2 (1.8, 2.6)		2.1 (1.7, 2.5)		1.7 (1.2, 2.3)		5.4 (5.0, 5.8)	
Interaction test		0.01*		0.062		0.001*		0.337		0.006*
**Residence permit in Lebanon**										
Yes	0.9 (0.8, 1.1)		1.8 (1.2, 2.4)		1.5 (0.8, 2.2)		1.0 (0.2, 1.7)		5.0 (4.4, 5.5)	
No	1.2 (1.1, 1.3)		2.0 (1.6, 2.4)		1.7 (1.3, 2.1)		1.9 (1.5, 2.4)		5.1 (4.7, 5.6)	
Interaction test		0.026*		0.519		0.614		0.035*		0.642

Statistically significant results are marked with an asterisk (*p* < 0.05). Abbreviations: SRH = Self-rated health. P = p-value. RR = Relative risk. CI = Confidence interval. B = beta coefficient

In Additional file 3, prevalence of good SRH and mean scores for the QoL domains at baseline and follow-up with stratification on variables showing statistically significant effect modification are reported. Here we can see that participants with low social support at baseline also had low levels of good SRH, psychological health, social relationships and environmental factors with a subsequent increase in each of these variables at follow-up.

Changes in the main outcomes did not differ by level of education, health literacy, time in transit or if migrating alone or with family (not shown in table).

## Discussion

Our study used longitudinal data to examine changes in SRH and QoL among Syrian refugees at two stages of their migration path. Overall, we found that SRH remained stable while QoL increased significantly in the short follow-up period of one year. Furthermore, our results suggest that gender, age and factors connected to the situation in transit (social support and residence permit in transit country) are important effect modifiers of change in SRH and QoL. The generally positive outcomes from this study lend credence to the notion of refugees’ inherent health resources stimulating growth and resilience [27]. A positive subjective health outcome is an essential means to successful integration, at the same time as successful integration enables good health [28].

Over half of the refugees rated their health as good at baseline (58%). This finding corresponds to levels of SRH measured in Syrian adults residing in pre-war Syria (55.3%) [29] and is also similar to previous findings on SRH among forced migrants resettled in high income countries, ranging from 58 to 64% [30, 31]. In contrast, in the general Norwegian population, over 70% rated their health as good [32]. Thus, we postulate that our cohort of forced migrants do not have an evident health advantage when compared with their final host population, which contradicts the healthy migrant effect/paradox [4, 6]. Notably, the SRH level increased marginally but non-significantly after only one year in resettlement.

Additionally, we found that the pre-arrival QoL scores for physical health, psychological health, and environment were rated significantly lower than the mean scores from the WHOQOL-BREF international field trials [26]. The physical and psychological domain improve significantly after resettlement but remain lower than international reference scores. In the environmental domain, mean QoL-scores surpass the levels of international reference scores after resettlement. Only a few previous studies have explored the concept of QoL specifically in forced migrants. Some of them found low scores in the environmental domain [33, 34] while others did not [35], but comparison is impeded by heterogeneity in the samples, apparent differences in migrant legal status and differences in countries’ reception schemes upon arrival. In our study, the lowest ratings at baseline were seen in the environment domain, which contains facets on financial resources, safety and security, accessibility of healthcare services and physical environment. Low scores could be attributed to circumstances observed in refugee settlements where unstable living conditions and poor provision of health services are prevalent. Our finding that all three domain scores: physical, psychological, and environment, improved after one year’s stay in the host country supports this theory. In addition, supportive resources upon arrival and favorable integration policies might have contributed to outweigh the effect of post-migration stressors [13].

The social relationship domain scores were lower than international reference scores at baseline but exceeded both international and Norwegian reference scores at follow-up [26]. Even though migration is a main cause of family disruption, most participants in our sample were resettled together with other family members, which might partially explain the high scores in social relationships. Some studies have reported favorable social relationships scores among forced migrants [35, 36] while others found results pointing in the opposite direction [37]. A high social capital has been identified as an important protective factor for poor mental health outcomes [38] and in sustaining refugee resilience and acculturation in the resettlement process [39]**.**

We found stronger improvement in SRH and two out of four QoL domains among men compared to women. These gender-related differences are comparable with evidence from previous research reporting worse health outcomes for female refugees [2, 30]. A gender-gap in SRH-measures has for long been conceptualized by researchers and has been attributed to a combination of biological and socio-behavioral differences [40]. In addition to known gender differences in SRH, the migration experience most likely affects men and women differently [3]. In the psychological domain, there was a larger improvement among younger participants, aged less than 40. This supports the notion of greater resilience seen in younger refugees [2, 41]. Moreover, we found that participants with low social support while in Lebanon had stronger improvement in SRH and QoL. Since there is a strong correlation between social support, SRH and QoL at baseline and their baseline measures were much lower than participants with high social support, this improvement indicates a larger “catch-up” for a group with an inferior starting point. It also means that within the right circumstances, an increase in SRH and QoL can be achieved regardless of your starting level of social support. The same catch-up phenomenon was seen for the ones who did not have a residence permit in Lebanon. Again, both these findings could point to internal resources in the refugee population enabling adjustment and growth after adversity. Contrary to our expectations, education - a social determinant of health, was not identified as a positive modifier of improvement. This could be attributed to the negative effect of losing your status prevailing over the protective effect of education [2]. Only a few migrated without family (16%) and it is possible that this small number made us unable to detect significant interactions for this variable.

### Strengths and limitations

The main strength of our study is the unique pre-arrival assessment that enabled us to trace refugee health outcomes before and after arrival to the host country using a longitudinal design. To our knowledge, this is a novel contribution to the research field allowing us to shed light on the sequential changes in health in a people moving from completely disparate settings. Secondly, we have a high response rate. In joint, the use of only validated instruments and a high response rate supports the internal validity of the study.

However, our findings should be interpreted in the context of the following limitations. Primarily, since there are no available registers on forced migrants during migration, we cannot state to which degree our sample is representative for the target population. This lack of an overall sample frame is a common limitation to observational studies on migrant health [42]. To compensate for this, efforts were put in the design to increase representativeness by inviting all the persons from Syria that were to be resettled to Norway in a given time period, as well as having a long recruitment period and recording of non-participation. Another limitation could be the deliberate change in assessment method from mainly self-completed questionnaire at baseline to telephone interviews at follow-up that introduces the possibility of interviewer bias. We used a short follow-up time that gives us important insight into the first phase of resettlement. However, we lack a long-term perspective. Prior research has shown deterioration in health over time [43] which warrants further longitudinal follow-up.

Our findings of an overall healthy cohort of refugees showing improvement in QoL in a short period of time provide important and novel information about a phase of the migration trajectory where little previous knowledge exists. From a clinical point of view, this information can encourage a shift in attention from pathogenesis to salutogenesis [44]. Recognizing positive health outcomes and refugees’ inherent health resources is important in the developing of interventions to bolster growth, resilience, and adaptation for the general refugee. In a policy-making setting, our findings suggest that women and older refugees should be subjected to a special effort to improve health. Our findings are also important in informing political and public discourse, nuancing the perception of refugees as a group with an inferior health status. We recommend more in-depth research to understand the mechanisms behind this rapid increase in QoL so that it can be sustained.

## Conclusion

We found stability in SRH and improvement in QoL in the early resettlement phase of refugees, more in younger age and among men compared to women. In addition, the social relationship and environment domain of QoL surpassed the levels of international reference scores after resettlement. Policy-makers and health care professionals should acknowledge that health of refugees is dynamic and can show rapid improvement after resettlement. To promote health equity and facilitate migration reception and integration, both short-term and long-term health outcomes should be taken into account.

## Supplementary information

**Additional file 1.**

**Additional file 2.**

**Additional file 3.**

## Data Availability

The datasets generated for the present study are not publicly available due to data protection regulations in Norway.
