# Supplementary figures and images for: Changes in self-rated health and quality of life among Syrian refugees migrating to Norway: a prospective longitudinal study
Source: Int J Equity Health. 2020 Oct 27;19:188. doi: 10.1186/s12939-020-01300-6 (PMC7590794; doi:10.1186/s12939-020-01300-6)

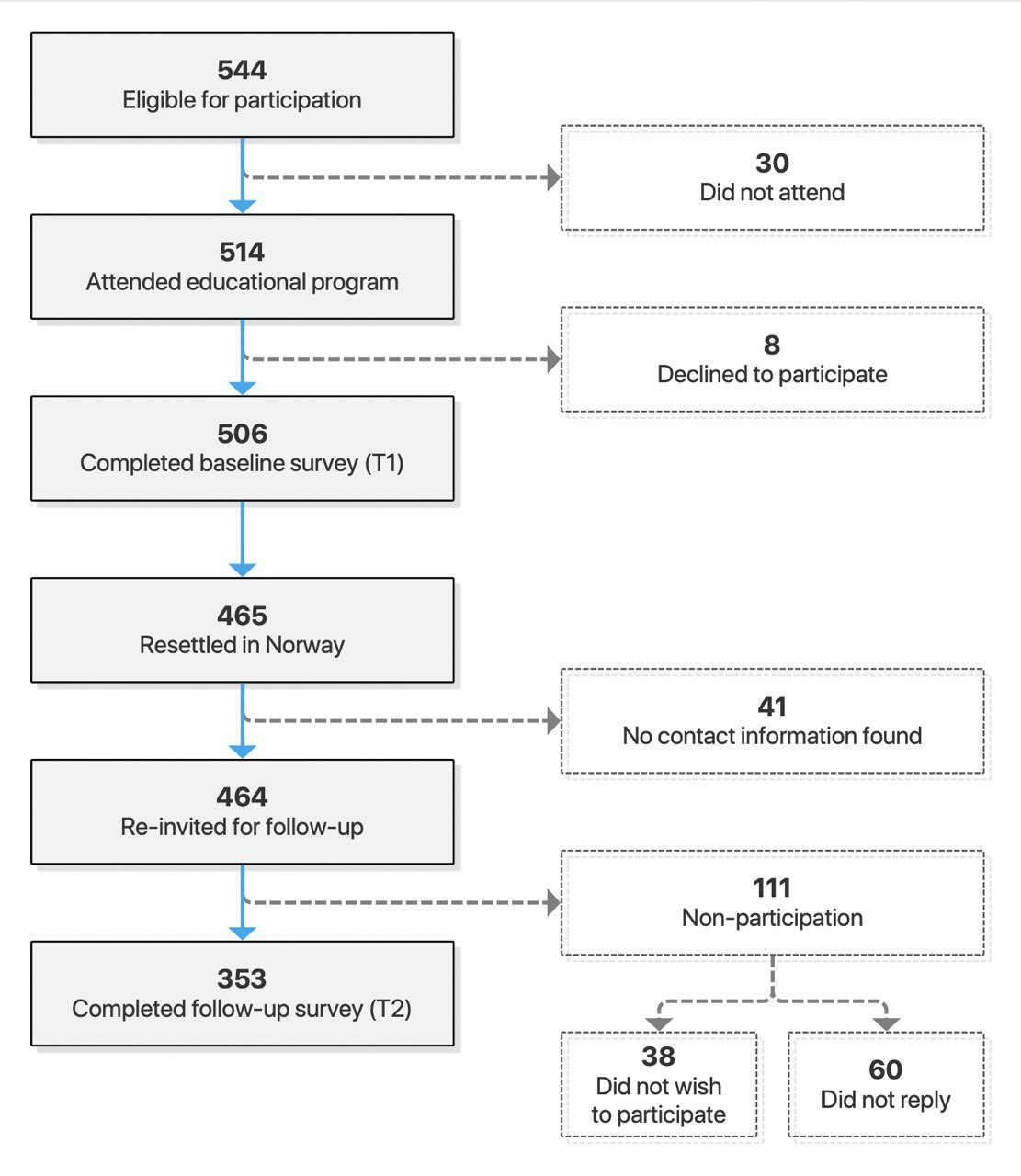

Supplement: Supplementary file 1 — Additional file 1. [file 12939_2020_1300_MOESM1_ESM.png]
