# Supplementary material for: Changes in self-rated health and quality of life among Syrian refugees migrating to Norway: a prospective longitudinal study
Source: Int J Equity Health. 2020 Oct 27;19:188. doi: 10.1186/s12939-020-01300-6 (PMC7590794; doi:10.1186/s12939-020-01300-6)
Supplement: Supplementary file 2 — Additional file 2. [file 12939_2020_1300_MOESM2_ESM.docx]

**Additional file 1. Flow-chart of recruitment process**

(see separate attachment to view figure) File format:

**Additional file 2. Sociodemographic and migration related factors among participants included at both measure points compared with loss to follow-up at second measure point**

|  | **Included at both time points** | **Lost to follow-up** | **P-value** |
| --- | --- | --- | --- |
|  | **N =353** | **N=153** |  |
| **SOCIODEMOGRAPHIC FACTORS** | | | |
| Gender (women), n (%) | 181 (51) | 74 (48) | 0.53 |
| Age (years), median (IQR) | 34 (27-41) | 36 (29-41) | 0.16 |
| Mother tongue, n (%) |  |  | 0.51 |
| Arabic | 335 (95) | 146 (95) |  |
| Kurmanji | 15 (4) | 7 (5) |  |
| Marital status, n (%) |  |  | 0.37 |
| Married | 265 (75) | 112 (73) |  |
| Living with partner among married | 260 (98) | 112 (99) | 0.37 |
| Number of children, median (IQR) | 3 (2-4) | 3 (2-4) | 0.44 |
| Education (years), median (IQR) | 8 (6-10) | 8 (6-10) | 0.54 |
| High health literacy | 195 (56) | 68 (45) | 0.02 |
| High social support | 123 (35) | 55 (36) | 0.81 |
|  |  |  |  |
| **MIGRATION RELATED FACTORS** | | | |
| Time since flight from Syria at baseline (years), median (IQR) | 5 (4-6) | 5 (4-5) | 0.01 |
| Time since arrival in Lebanon at baseline (years), median (IQR) | 5 (4-5) | 5 (4-5) | <0.01 |
| Been in other transit country before Lebanon, n (%) | 20 (6) | 10 (7) | 0.72 |
| No residence permit in Lebanon at baseline, n (%) | 242 (69) | 95 (63) | 0.16 |
| Migrating alone to Lebanon, n (%) | 55 (16) | 20 (13) | 0.44 |

Abbreviations: IQR = interquartile range
