# Supplementary material for: Changes in self-rated health and quality of life among Syrian refugees migrating to Norway: a prospective longitudinal study
Source: Int J Equity Health. 2020 Oct 27;19:188. doi: 10.1186/s12939-020-01300-6 (PMC7590794; doi:10.1186/s12939-020-01300-6)
Supplement: Supplementary file 3 — Additional file 3. [file 12939_2020_1300_MOESM3_ESM.docx]

**Additional file 3. Changes in prevalence in dichotomous outcome (SRH) and mean (SD) score for continuous outcome (WHOQOL-BREF four domain scores, range 4–20) from baseline to follow-up stratified on selected sociodemographic and migration related factors, N= 353**

|  | **Good SRH** | | **Physical health** | | **Psychological health** | | **Social relationships** | | **Environment** | |
| --- | --- | --- | --- | --- | --- | --- | --- | --- | --- | --- |
|  | **T1 (%)** | **T2 (%)** | **T1 (mean)** | **T2 (mean)** | **T1 (mean)** | **T2 (mean)** | **T1 (mean)** | **T2 (mean)** | **T1 (mean)** | **T2 (mean)** |
| **Gender** |  |  |  |  |  |  |  |  |  |  |
| Male | 56 | 67 | 13.6 | 15.8 | 12.8 | 14.7 | 13.5 | 15.6 | 8.7 | 14.2 |
| Female | 60 | 59 | 13.8 | 15.5 | 12.8 | 14.3 | 13.8 | 15.0 | 9.2 | 13.9 |
| **Age** |  |  |  |  |  |  |  |  |  |  |
| < 40 years | 63 | 68 | 13.8 | 16.0 | 12.8 | 14.7 | 13.7 | 15.3 | 9.0 | 14.1 |
| ≥ 40 years | 44 | 48 | 13.4 | 14.7 | 13.2 | 14.0 | 13.7 | 15.1 | 8.9 | 13.9 |
| **Marital status** |  |  |  |  |  |  |  |  |  |  |
| Married | 53 | 59 | 13.5 | 15.5 | 12.7 | 14.5 | 13.8 | 15.3 | 8.7 | 14.0 |
| Other | 75 | 76 | 14.4 | 16.0 | 13.4 | 14.6 | 13.2 | 15.3 | 9.7 | 14.2 |
| **High social support (ESSI)** |  |  |  |  |  |  |  |  |  |  |
| Yes | 64 | 58 | 14.1 | 15.6 | 13.7 | 14.5 | 14.4 | 15.7 | 9.6 | 14.1 |
| No | 55 | 66 | 13.5 | 15.7 | 12.4 | 14.5 | 13.3 | 15.0 | 8.6 | 14.0 |
| **Residence permit in Lebanon** |  |  |  |  |  |  |  |  |  |  |
| Yes | 66 | 62 | 14.0 | 15.8 | 13.2 | 14.8 | 14.1 | 15.0 | 9.1 | 14.1 |
| No | 55 | 65 | 13.6 | 15.6 | 12.7 | 14.4 | 13.5 | 15.4 | 8.9 | 14.0 |

T1 = first measure point (baseline). T2 = second measure point (follow-up)
